# Edges in brain networks: Contributions to models of structure and function

**DOI:** 10.1162/netn_a_00204

**Published:** 2022-02-01

**Authors:** Joshua Faskowitz, Richard F. Betzel, Olaf Sporns

**Affiliations:** Program in Neuroscience, Indiana University, Bloomington, IN, USA; Department of Psychological and Brain Sciences, Indiana University, Bloomington, IN, USA; Indiana University Network Science Institute, Indiana University, Bloomington, IN, USA; Cognitive Science Program, Indiana University, Bloomington, IN, USA

**Keywords:** Connectome, Network, Edge, Structure function relationship, Connectivity, Network construction, Network communication

## Abstract

Network models describe the brain as sets of nodes and edges that represent its distributed organization. So far, most discoveries in network neuroscience have prioritized insights that highlight distinct groupings and specialized functional contributions of network nodes. Importantly, these functional contributions are determined and expressed by the web of their interrelationships, formed by network edges. Here, we underscore the important contributions made by brain network edges for understanding distributed brain organization. Different types of edges represent different types of relationships, including connectivity and similarity among nodes. Adopting a specific definition of edges can fundamentally alter how we analyze and interpret a brain network. Furthermore, edges can associate into collectives and higher order arrangements, describe time series, and form edge communities that provide insights into brain network topology complementary to the traditional node-centric perspective. Focusing on the edges, and the higher order or dynamic information they can provide, discloses previously underappreciated aspects of structural and functional network organization.

## INTRODUCTION

Modern neuroscience has come to appreciate the complexity of the brain’s wiring structure and functional dynamics. Increasingly, neuroscientists employ the tools of network science to model the brain as a network, a mathematical representation of data well suited to investigate complex systems (Bassett & Sporns, 2017; Bullmore & Sporns, 2009). Brain networks can reveal many aspects of brain structure and function, including clusters and modules (Betzel, Medaglia, & Bassett, 2018), or information flow and communication (Avena-Koenigsberger, Misic, & Sporns, 2018). Approaching the brain as a network, a connectome (Sporns, Tononi, & Kotter, 2005) composed of distinct elements and their interrelationships, naturally integrates local and global perspectives, linking the roles of individual network elements to distributed function.

There are many ways to map and represent connectomes. For a select few “model” organisms, the microscale, single-neuron networks of the compete nervous system have been meticulously documented *via* electron microscopy (White, Southgate, Thomson, & Brenner, 1986). Other approaches, using techniques that afford less spatial resolution while offering broader coverage, have yielded meso and macroscale connectomes across many species, including humans. For example, noninvasive imaging allows the brain to be represented as a network of inferred paths of axonal tracts through the white matter (Hagmann et al., 2008), of morphometric similarity between parts of the cortex (Seidlitz et al., 2018), or of functional correlation of intrinsic hemodynamic fluctuations across time (Biswal et al., 2010). Brain networks provide a universal modeling framework enabling comparisons across data modality, scale, and species.

The nodes of brain networks are generally taken to represent distinct neural elements, such as neurons, neuronal populations, or regions, while the edges record the dyadic (pairwise) relationships between these elements. Fundamentally, these two components of the network model are inseparable. Nodes would not connect without edges, and edges would be ill-defined without nodes. Yet, when applied to the brain, network models often prioritize nodes, describing and differentiating their mutual relations and functional contributions. Examples of key “node-centric” concepts are highly connected hubs, which integrate information, or densely connected, communities associated with specialized functional systems. Furthermore, networks are often globally described through distributions of measures like node degree, strength, clustering, or participation coefficient, and the network’s community structure is almost exclusively expressed as nodal partitions. Finally, node metrics are frequently used to probe for associations with behavioral or genetic traits. The focus on the nodal characteristics extends prevailing trends in the long history of brain mapping, which has been dominated by the search for localized neural elements that relate to specific functions (Raichle, 2009). Less heralded are the edges. While providing crucial information to make these nodal network assessments, they are rarely seen as primary descriptors of brain network organization.

Even though edges are half of the network model, many issues concerning the brain’s interrelationships have so far been underappreciated. The edges of the brain, and their collective topology, are key ingredients that transform and elevate static maps of the brain (“wiring diagrams”) into distributed and dynamic systems capable of supporting behavior and cognition. Not only do edges play a role in characterizing the direct links between functionally meaningful regions, but taken together, they also form distributed patterns that further characterize the brain’s complexity. Here, we shine a spotlight on brain network edges, surveying the ways in which information located between the nodes can be used to understand brain network organization. We begin by clarifying that the type of edge, supported by underlying neural data, is consequential for the downstream network analyses. Then, we review the various constructs that edges can jointly form, which are useful because they can capture relationships that extend beyond pairwise interactions. We cover the importance of edges for studying brain communication and briefly review ways in which communication dynamics evolve over time at the edge level. Finally, we look to the future, and include a discussion of several new developments for interpreting information at the edge level. Overall, we endeavor to bring attention to the importance of brain network edges and to demonstrate the value in carefully considering the information they provide.

## NETWORK CONSTRUCTION

Networks offer a universal language to describe complex systems made up of many interacting parts. The basic ingredients for any network are its nodes and edges. The nodes describe the discrete elements of a system, whereas the edges express the relationships that can be measured between these elements. While the definition of networks as sets of nodes and edges is universal, which real-world constructs are taken to be nodes and which as edges depends on assumptions and interpretations that guide the construction of the network model (Butts, 2009). Depending on the system being modeled, edges may be binary or may carry a weight. Weights may be both positive and negative, and they may express directed or undirected relations. In many real-world networks, like a social network, the subway map, or a power grid, these basic network ingredients are generally well defined and accessible to data collection. In contrast, defining the nodes and edges of a brain network is less straightforward.

**Figure 1.**
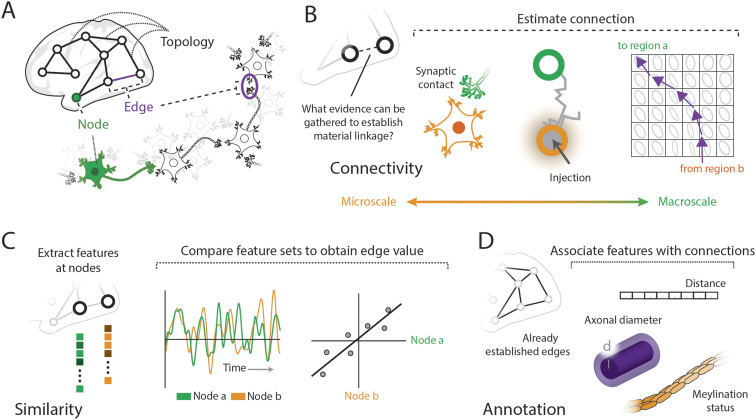
The relational content of the brain can be documented in several manners. (A) The basic components of a brain network, the *nodes* and *edges*, can be modeled across scales, spanning neurons to cortical regions. (B) Edges of connectivity report the ways in which nodes can be materially linked, across spatial scales; at the microscale, these edges can represent neuronal contact whereas at the macroscale, such edges can be estimated via computational processes like tractography. (C) Edges of similarity report the ways in which feature sets at nodes are alike; such feature sets can be gathered from both dynamic and static data. (D) Edges can be annotated with weights from other modalities or embeddings, adding an additional layer of information on the network.

Aside from the microscale, where it could be argued that nodes and edges unambiguously correspond to neurons and synaptic contacts (Figure 1A), representing brain data as a network requires choosing from a wide range of node definitions as well as picking a valid mode and metric for their interrelationships (Bassett, Zurn, & Gold, 2018). As such, it has been demonstrated that definition of nodes and nodal parcellations can significantly influence the results of downstream network analyses (Arslan et al., 2018; Messe, 2020; Zalesky et al., 2010). Edge definition is just as consequential. Focusing on the brain’s interrelationships, we can broadly classify edges as documenting connectivity or similarity between the brain’s nodes. Additionally, edges can be annotated with supplemental measurements or carry weights that reflect the fusion of multiple modalities (see Box 1).

**Box 1.**  Alternative weighting strategies

Measurements of attributes that annotate existing edges can also be taken between neural elements (Figure 1D). Edges of similarity and connectivity provide a quantification of the relationship between two nodes and collectively define the topology of a given brain network. Already existing or estimated edges can be associated with metrics representing additional features, possibly derived from another modality or an embedding space. This approach allows for network edges to carry annotated layers of data derived from sources not directly related to the network construction process. Such features can aid computational modeling or data analysis. Attributes such as Euclidean distance, tract length, conduction delays, axonal caliber, biophysical efficacy, connection cost, or indices of myelination status are all examples of attributes that can be ascribed to edges expressing connectivity or similarity.

**Figure 2.**
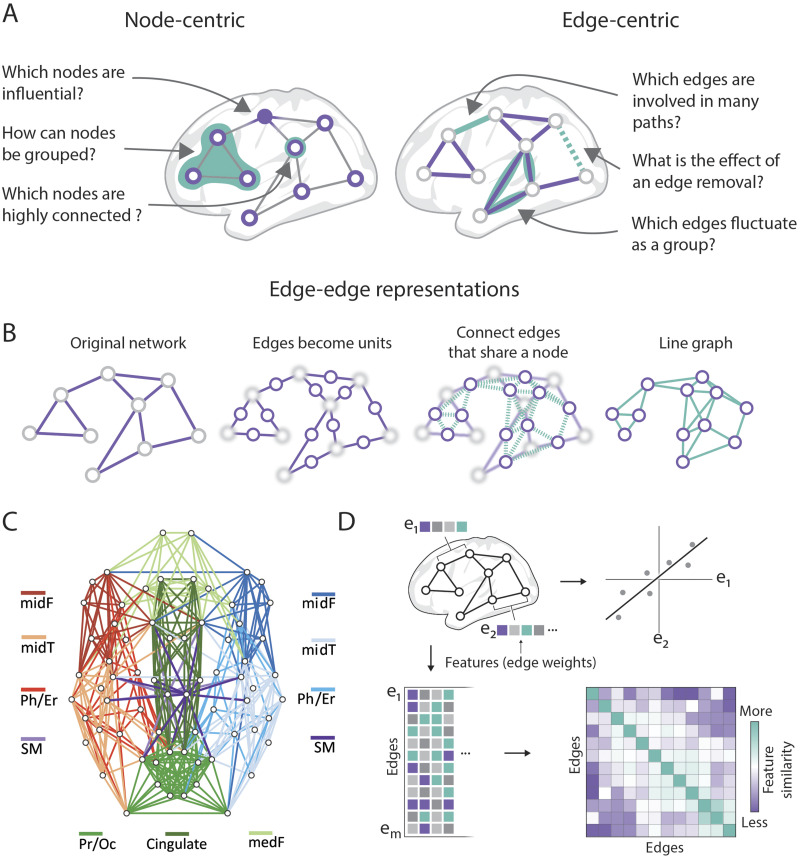
Network science offers a wide range of tools and methods to analyze brain networks. (A) Many common brain network analyses extract information about the nodes (i.e., node-centric), such as their centrality or modular groupings; edge-centric brain network analyses can annotate the edges with information, such as the proportion of shortest paths that pass through an edge or the effect of an edge’s removal on global network statistics. (B) Line graphs are representations of a network that capture how edges are connected to each other *via* nodes, as depicted in this diagram. (C) Clustering an edge-edge network representation, in which network incidence (e.g., line graph) or pairwise edge similarity is assessed, results in an edge community structure; by affiliating each edge with a cluster, each node is associated multiple (or overlapping) communities (figure reproduced from M. A. de Reus, Saenger, Kahn, & van den Heuvel, 2014, with permission from The Royal Society, UK). (D) The pairwise similarity between edges can be assessed by correlating feature sets at the edges, such as multiple tractography streamline weights or functional correlation measures taken during distinct tasks (figure adapted from Faskowitz, Tanner, Misic, & Betzel, 2021).

Edges can also be annotated with a value that reports a summary statistic or the result of combining several relational measures into a single weight. In this way, edges can carry weights that report a relationship generated from several modalities or conditions. Take morphometric similarity, for example, which reports the correlation of standardized indices of myelination, gray matter, and curvature taken at the nodes (Seidlitz et al., 2018). The edge weight here reflects a similarity across imaging domains that assess different aspects of the cortical geometry and composition. Within the realm of functional imaging, a generalized measure of functional coactivity between nodes can be estimated by combining data from rest and task sessions (Elliott et al., 2019). Such a procedure can increase the reliability of intrinsic connectivity estimation. Relatedly, correlation values from various scan sessions can form a feature set at each edge (Figure 2D), which can be used to create an edge-centric representation of edge covariance across conditions (Faskowitz et al., 2021). Thus, edges can report multifaceted relationships incorporating a variety of data sources.

### Edge Types: Connectivity

Edges can represent connectivity between neural elements, quantifying material linkage or contact, supporting information flow, signal spread, or communication, and summarized in a sparse connectivity or adjacency matrix (Figure 1B). Depending on data modality, connectivity can be resolved from the micro- (White et al., 1986) to the macroscale (Hagmann et al., 2008), providing varying levels of evidence of a true (physical) connection. At the microscale, edges represent synapses or gap junctions, resolved with techniques such as electron microscopy or through light-microscopic labeling and imaging (Motta et al., 2019). At increasing scales, neural data documents coarser patterns of connectivity that link populations of neurons comprising one or more cell types or layers or representing entire brain regions. In mammalian brains, such interregional connections are often myelinated, collectively forming the brain’s white matter, and can be mapped with a variety of techniques. For example, tract tracing is used to label and reconstruct interregional projections (Markov et al., 2014; Oh et al., 2014). Generally, multiple reconstructions need to be combined to achieve robust characterization of connection patterns and weights. One approach is to informatically collate the literature of tract tracing experiments, to create comprehensive maps that also record ordinal assessments of connection weights (Bota, Sporns, & Swanson, 2015; Kotter, 2004). At the scale of millimeters, bundles of topographically organized axonal paths through the white matter, commonly referred to as tracts, can be estimated via tractography (Jbabdi, Sotiropoulos, Haber, Van Essen, & Behrens, 2015) and serve to quantify connectivity (Sotiropoulos & Zalesky, 2019; Yeh, Jones, Liang, Descoteaux, & Connelly, 2020). Common to these edge definitions expressing connectivity is a notion of anatomical substrate enabling various patterns of between-node communication. A different approach aims to infer patterns of effective connectivity that correspond to causal relationships and influences (Friston, 2011). Effective connectivity is estimated from functional data via methods that establish statistical or model-based causality between time-varying nodal signals (Reid et al., 2019; Valdes-Sosa, Roebroeck, Daunizeau, & Friston, 2011) or perturbational evidence (Lim et al., 2012). Ultimately, edges of connectivity define the potential for one node to influence another, made possible by estimated anatomical linkage.

### Edge Types: Similarity

Edges can also denote the similarity between node-level features (Figure 1C). Computing the statistical similarity (or distance) between each pair of nodal feature sets forms a dense similarity matrix (all entries are nonzero), which may be interpreted as a network. Notably, the feature sets at each node reflect data points collected across space or time, which modulates the interpretation of such edges. Using imaging or histological observations, neuroanatomical features can be sampled at each node, including for example cortical thickness (Carmon et al., 2020) or layer intensity profile (Paquola et al., 2019). These features can then be statistically compared within or across subjects (Alexander-Bloch, Giedd, & Bullmore, 2013) to create edges that represent the similarity of feature sets. The interpretation of similarity-based edges varies depending on what is included in the feature set. For instance, structural similarity, which may reflect cytoarchitectonic similarity, is thought to relate to anatomical connectivity (Goulas, Majka, Rosa, & Hilgetag, 2019). Another similarity-based approach quantifies correlated gene expression between areas of cortex (Richiardi et al., 2015), made possible by extensive brain atlases documenting genetic profiles in stereotaxic space (Hawrylycz et al., 2012; Ng et al., 2009). Edges based on correlated gene expression among a set of genes known to be enriched in supragranular cortex align with canonical system organization (Krienen, Yeo, Ge, Buckner, & Sherwood, 2016) and show significant association with edges of structural covariance (Romero-Garcia et al., 2018). Finally, the informatic collation of functional activation experiments provides across-study evidence that certain region pairs coactivate more readily than others, forming meta-analytic coactivation edges (Crossley et al., 2013).

Recordings of activity time series at neural elements may be taken to represent temporally resolved feature sets whose similarity, or more generally, statistical association, is widely employed to interrogate brain organization. Neural activity can be recorded across a range of resolutions and frequencies and, in turn, can serve as the basis of many types of bivariate similarity calculations (Smith et al., 2011; see also Basti, Nili, Hauk, Marzetti, & Henson, 2020). Neural recordings with high temporal precision, such as electrical potentials or magnetic fields (Hari & Puce, 2017), provide data allowing the resolution of directed, non-linear, and/or information theoretic edge weights (Astolfi et al., 2007; Ince et al., 2017). Brain signals recorded at lower temporal resolution, such as the blood oxygen level–dependent (BOLD) signal or Ca^2+^ recordings, can be compared using Pearson correlation or wavelet coherence. Such edges are generally referred to as “functional connectivity” (Friston, 2011), essentially encapsulating the collective node dynamics in the form of a covariance matrix (Reid et al., 2019). A looming topic in studies of functional connectivity is that of the dynamics of functional relationships, and if observed fluctuations in similarity represent neurobiologically relevant processes or mere statistical variance in an otherwise stationary relationship (Laumann et al., 2017; Lurie et al., 2020). Relatedly, the similarity of dynamics could be influenced by cognitive state, raising the question whether the recorded edge represents a trait or state measurement (Geerligs, Rubinov, Cam, & Henson, 2015). Dynamics at each node can also be used to collect large feature sets of time series properties (Fulcher & Jones, 2017), which can be used to compare temporal profile similarity (Shafiei et al., 2020), an edge measure that is distinct from correlation and can reveal dynamical hierarchies.

## EDGE-CENTRIC NETWORK ANALYSES

Once a brain network is constructed, common practice is to use the tools of network science and graph theory to describe the organizational patterns of the data (Fornito, Zalesky, & Bullmore, 2016; Rubinov & Sporns, 2010). In many instances, network analyses are used to obtain information about nodes, asking questions like: Which nodes are most influential, or highly connected? How can these nodes be meaningfully grouped (Figure 2A)?

Network analyses that result in information at the edge level provide complementary insights (Figure 2A). A common edge construct is the path, an ordered sequence of unique edges that links a source to a target node. Edgewise metrics based on paths include the edge betweenness centrality which describes the fraction of shortest paths that traverse a specific edge. Paths are important for network communication as they define possible routes for information flow. Communication models use network paths (“routing”) or random walks (“diffusion”) to estimate the potential for communication between nodes, resulting in a dense communication matrix where each edge expresses a valuation of this potential (Goni et al., 2014; Seguin, Tian, & Zalesky, 2020). Finally, the vulnerability of networks can be assessed by removing network components and observing the resulting effect (Henry et al., 2020). The simulated removal of specific edges can, for example, modulate readings of the network's topology (Ardesch et al., 2019; M. A. de Reus et al., 2014). This “edge-lesioning” approach can be applied to a range of common network measures, including those that produce measurements per node like clustering coefficient, and hence can assess the global effect of edge removal.

Network science also offers approaches to represent a *network of edges*, to focus on how the edges relate to each other (Figure 2B–2D). One approach is to construct a line graph which documents how edges share nodes (Figure 2B). Whereas a traditional network documents adjacency, or how nodes are linked via edges, a line graph documents incidence, or how edges are linked via common nodes (Evans & Lambiotte, 2009). For the line graph network representation, the network is essentially flipped inside out, with edges from the original network becoming nodes. In practice, the line graph has matrix dimensions of *E*-by-*E*, where *E* is the number of unique edges of the original network. A notable property of line graphs is that high-degree nodes (hubs) in the original network become dense clusters (cliques) in the line graph. Networks of edges distinct from line graphs can also be obtained by computing edge-similarity matrices. For example, an *E*-by-*E* similarity matrix may be obtained using the Jaccard index applied to edges (Ahn, Bagrow, & Lehmann, 2010). Clustering such edge-similarity matrices, or any *E*-by-*E* matrix, results in edge communities. These communities give rise to overlap at the level of nodes, where each node can be affiliated with multiple communities assigned to its emanating edges. Clustering a line graph of structural connectivity reveals bilateral spatially coherent link communities (Figure 2C), with differential connectivity scores per community, and community overlap that converges on nodes that are traditionally considered hubs (de Reus et al., 2014).

Networks are a universal phenomenon, and generally, the algorithms we apply to networks to uncover clustered, community, or scale-free organization are data agnostic. This means that network measures like the clustering coefficient are easy to compute on a power grid, a brain network, or any other sort of network in hand with a minimal set of assumptions (fulfilling the requirements of a *simple graph*, a network without self-loops and hyperedges). However, while it is possible to run the gamut of network tools on brain data, doing so without considering the source of the neural data and the ensuing interpretation of nodes and edges is unwise. The incorporation of domain-specific neuroscience expertise—knowledge about the neural data source, and an understanding of how a network measure relates to the aspect of brain organization being modeled—should be a key consideration when analyzing brain networks.

Edges in brain networks can be defined in different ways. Importantly, information about how an edge was constructed and the underlying relationship that the edge is intended to represent affects how the network should be analyzed. Take for example path-based measurements applied to brain networks. Paths over structural edges are intuitive and have physical meaning, given that a path may represent hypothetical signal propagation over a material substrate (Avena-Koenigsberger et al., 2017; Mišić et al., 2015). For such structural paths, its constituent edges and edge weights should reflect the cost or capacity of communication between nodes, such as distance, speed, volume, or bandwidth.

Paths over functional edges that express similarity are less intuitive, and possibly ill-conceived, compared to paths over edges of connectivity. What does a path over functional similarity measurements mean? One possible argument is that structural and functional edge weights are indeed positively associated (Honey et al., 2009), so that paths over functional similarities may, to some extent, be associated with underlying connectivity. However, given that measures such as Pearson correlation express mixtures of direct and indirect sources of variance in a networked setting (Sanchez-Romero & Cole, 2021; Zalesky, Fornito, & Bullmore, 2012), this interpretation is likely too charitable. Another approach for using functional edges to construct paths is to study the transient routes that appear along the underlying structural graph (Griffa et al., 2017). Network paths and their derived measures should be interpreted differently based on edge type, as they likely capture different organizational features of a brain network.

Another instance in which the edge definition influences network analysis is the case of surrogate data modeling, when an empirical network measurement needs to be compared to hypothetical, yet plausible, network topologies. Null models should be able to create surrogate data that recapitulate certain network characteristics, like a similar degree distribution, but with a different pattern of edges (Betzel et al., 2016a; Faskowitz & Sporns, 2020; Rubinov, 2016). Generally, null models are important for evaluating the significance of descriptive network statistics by providing plausible network configurations to benchmark against. Additionally, null models are used to help infer network organization, like in the application of modularity maximization, which searches for clustered edge weights above a baseline rate commonly estimated with an edge-swapping null model. However, for brain networks constructed from statistical comparisons, there exist more suitable null models that account for signed edges (Rubinov & Sporns, 2011) or spatial information (Esfahlani et al., 2020) and take into account the transitive relationships between edges (Zalesky et al., 2012). An unrealistic null model could be insensitive to certain biases, such as the distance-dependence of edges (Choi & Mihalas, 2019), which in turn can alter the inferred network organization (Betzel et al., 2017). In applications of community detection and beyond, null models that account for the physical distance distribution of edges are a more accurate model of the brain, which is spatially embedded (Horvat et al., 2016; Roberts et al., 2016) (see Box 2). Surrogate data that does not account for the distance distribution of edges will be less efficiently embedded, with longer connections than expected (Bassett et al., 2010). For network neuroscience, null models broadly fall into two categories. Generative null models describe the placement or formation of edges between nodes, often based on simple rules that after repeated application, can form complex topology (Akarca et al., 2021; Vertes et al., 2012). Rewiring null models alter the given topology of a network by swapping or reweighting edges, according to specified constraints or rules (Kaiser & Hilgetag, 2006; Roberts et al., 2016). Network science offers a range of null models which neuroscientists can choose from or modify, to better align with edge definition (Fornito et al., 2016).

**Box 2.**  Spatial embedding makes brain networks unique

Networks are models of interrelationships between a system’s elements. In many systems, there is no inherent cost to forming a connection. Consider the world wide web, in which nodes and edges represent URLs and hyperlinks, respectively. The “cost” of adding a hyperlink from one URL to another is minimal in that it requires no material contribution and (apart from the physical energy associated with writing HTML code) entails no metabolic or energetic expense. The lack of any explicit cost is a direct result of the fact that the WWW is not embedded in a physical space. The human brain, in contrast, is embedded in Euclidean space where the axonal projections and white-matter tracts require material to be formed and energy to be maintained and used for signaling (Stiso & Bassett, 2018). For physical systems like the brain, forming and maintaining a network is costly. From a network’s perspective, these costs are felt at the level of edges, where material and metabolic costs depend on geometric characteristics of anatomical connections, for example, their length and diameter (Rivera-Alba et al., 2011).

Brain networks are organized to reduce their material and metabolic expenditures, preferring to form short-range (and therefore less costly) connections. This preference, in turn, shapes the organization of the network and induces architectural features. For instance, networks that depend strongly on spatial constraints are naturally more clustered and readily form modules, making it difficult from an algorithmic perspective to adjudicate between “true” modules and those that reflect the underlying spatial constraints (Rubinov, 2016; Samu, Seth, & Nowotny, 2014).

On the other hand, brain networks do not strictly minimize their cost, forming a small number of long-distance connections (Betzel et al., 2016b; Roberts et al., 2016). Presumably, these connections confer a functional advantage to the brain, otherwise we would expect evolution to have replaced them with shorter (and less costly) connections. What roles do these costly long-distance connections play? In binary networks, they form “shortcuts” that reduce the network’s characteristic path length and enhance communication efficiency (Kaiser & Hilgetag, 2006). They also link high-degree nodes to one another, forming a constellation of interconnected hub nodes known as a “rich club,” which plays a key role in the integration of information from different systems (van den Heuvel & Sporns, 2011; Zamora-Lopez, Zhou, & Kurths, 2010). In weighted networks, however, long-distance connections play a reduced role due to their proportionally weaker weights (in spatial networks, connection weight tends to decrease monotonically with length). What role might these connections play? Across phylogeny, long-distance connections are both highly specific and robust, forming multiple bridges between the same distant neighborhoods. Recent work has suggested that these connections introduce unique and dissimilar signals into those neighborhoods, enhancing functional diversity and promoting increasingly complex dynamics (Betzel & Bassett, 2018).

Many observable real-world networks are sparse, in that relatively few edges exist out of all the possible pairwise node combinations. Estimates of structural connectivity between nodes are also observed to be sparse, particularly at finer spatial resolution and greater distances, possibly an outcome of selection pressure on wiring cost (Bullmore & Sporns, 2012). In contrast, similarity assessments result in fully dense networks that present practical and conceptual challenges for network analyses. Some practitioners may opt to selectively remove edges below a threshold to enforce sparsity (Fallani, Latora, & Chavez, 2017; Garrison, Scheinost, Finn, Shen, & Constable, 2015), with thresholds chosen according to across-group consensus (Betzel, Griffa, Hagmann, & Misic, 2019; van den Heuvel et al., 2017) or to retain a network feature such as a connected component or minimum spanning tree (Nicolini, Forcellini, Minati, & Bifone, 2020; Tewarie, van Dellen, Hillebrand, & Stam, 2015). Thresholding can induce biases and confounds (Zalesky et al., 2012) in the overall network topology and therefore must be performed with justification and with an understanding that different thresholds could possibly affect the investigation’s main findings. Alternatively, analytical approaches that incorporate noisy edges or imperfect graph observation could be a fruitful future direction for network neuroscience (Young, Cantwell, Newman, & Peixoto, 2020).

## EDGE CONSTRUCTS: FROM MOTIFS TO HIGHER ORDER RELATIONS

Edges on their own report a straightforward relational quantity. These quantities can be treated as elementary network features, to be associated with traits and behaviors through mass univariate testing, in what is sometimes referred to as a bag-of-edges approach or brain-wide association (Chung et al., 2021). However, edges may also be grouped together to form richer constructs that capture distributed patterns of brain organization. Small groups of edges form constructs that can be analyzed as building blocks or primitives of the complete network. Mass univariate methods could fail to uncover these higher order relationships, and even prove to be underpowered (Zalesky, Fornito, & Bullmore, 2010), because they focus on edges as independent entities. Here, we describe edge-based constructs moving from more localized patterns such as motifs or connectivity fingerprints to more global patterns of brain network topology.

### Motifs

**Figure 3.**
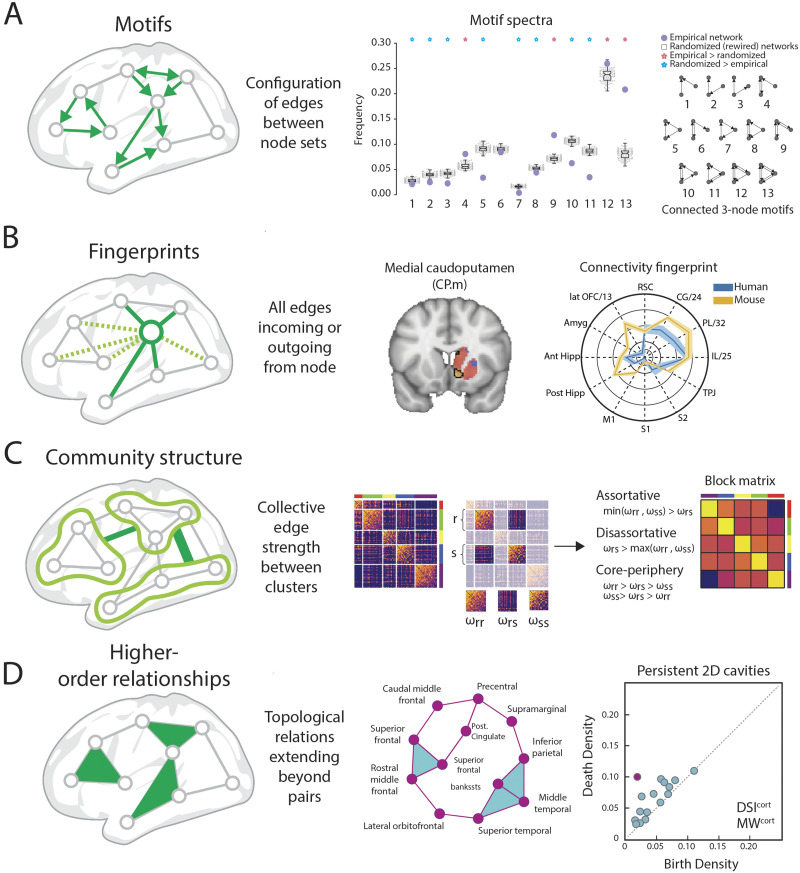
Edges can be grouped to form constructs amenable for analysis. (A) Motifs are characterized by a set number of nodes and the pattern of edges that fall between them; the motif spectra visualizes the frequency of various motifs present in the network (figure adapted from Z.-Q. Liu, Zheng, & Misic, 2020). (B) Connectivity fingerprints describe the set of edges connected to a specific node, which can create a global context or profile for a specific region and can be used to identify homologs across species (figure adapted from Balsters, Zerbi, Sallet, Wenderoth, & Mars, 2020). (C) Community structure describes a mesoscale organization of the network, which can be used to calculate and classify edge strengths between clusters (figure adapted from Betzel et al., 2018). (D) Higher order relationships, such as cliques and cavities, can be built by aggregating pairwise relationships to assess higher dimensional structure of the network (figure adapted from Sizemore et al., 2018).

Network motifs are subgraphs with a fixed number of nodes and differentiated by the pattern of edges falling between these nodes (Figure 3A). For example, between three connected nodes, there are 13 topologically unique ways that edges (directed and unweighted) can be placed, forming 13 motifs (Figure 3A). The frequency of that each motif’s expression tells us about the network’s local building blocks (Dechery & MacLean, 2018; Sporns & Kotter, 2004). Motif frequencies are assessed using surrogate networks, to gage the under- or overexpression of certain motifs (Horvat et al., 2016; Z.-Q. Liu et al., 2020) or can be related to principal dimensions of network organization (Morgan, Achard, Termenon, Bullmore, & Vertes, 2018). The edge configurations of specific motifs constrain the possible patterns of dynamic interactions (Sporns & Kotter, 2004) and enable temporal coherence and synchrony. For example, motif configurations containing bidirectional connections, termed resonance pairs, can induce zero-lag synchrony in a variety of neuronal spiking models, despite nonzero conduction delays on individual edges (Gollo, Mirasso, Sporns, & Breakspear, 2014). Taken together, network motifs express intermediate aspects of brain architecture and are thus informative for investigating how the wider network might support functional activity.

### Connectional Fingerprints

In virtually all brain networks, the pattern of incoming and outgoing edges attached to each node is unique. These edge patterns, known as connectional fingerprints (Figure 3B), were proposed as fundamental structural profiles that shape the functional specialization of a given region by determining from whom that region receives its inputs and to whom its outputs are delivered (Mars, Passingham, & Jbabdi, 2018; Passingham, Stephan, & Kotter, 2002). The fingerprinting approach can help to clarify the functional roles regions might play, based on their differential weights to other areas (Tang et al., 2019), or to predict functional activation patterns (Osher et al., 2016; Saygin et al., 2016). A key concept of the fingerprinting approach is the embedding of areas within an abstract connectivity space, as opposed to a geometric space (Mars et al., 2018). The connectivity space can be used, in conjunction with common structures, to help identify homologies between species (Balsters et al., 2020). Furthermore, this connectivity space can be used to subdivide larger regions based on fine-grained connectivity profiles (Genon et al., 2018).

From a network perspective, a connectivity fingerprint is a row or column of the adjacency matrix that records a vector of edge weights attached to each node. Notably, this row of edge weights is a discrete analog of traditional seed-based connectivity. The similarity of edge patterns can be measured using the normalized matching index (Fornito et al., 2016) or cosine similarity (Betzel & Bassett, 2018), to gage connectional homophily between nodes, which is a critical ingredient for generative models of brain networks (Betzel et al., 2016a). Ultimately, the pattern of edges emanating from each node describes the context of the node within the larger network architecture. The connectivity fingerprinting approach demonstrates the utility of assessing a complete pattern of connections to each node, rather than looking at only a subset.

### Community Structure

Although network communities are often interpreted from a node-centric perspective—most commonly defined as groupings of densely connected nodes—it is the edges that determine which nodes should be grouped together, whether by strength of connection (Sporns & Betzel, 2016) or by similarity of edge connectivity patterns (Faskowitz, Yan, Zuo, & Sporns, 2018; Moyer et al., 2015). Given an established or inferred community structure, the edges that fall between communities are used to characterize the integrative hublike roles of select nodes. For example, edge information is used to identify nodes whose edges are highly dispersed among functional areas (Bertolero, Yeo, & D'Esposito, 2015) or to classify hub areas associated with different cognitive domains (Gordon et al., 2018). Furthermore, the community structure can be used to reduce the network to its block structure, by recording the summed or averaged edge strength between communities (Figure 3C). This block structure characterizes mesoscale between-community connection patterns, such as modular, core-periphery, or disassortative configurations (Betzel et al., 2018; Faskowitz & Sporns, 2020).

### Higher Order Relationships

Thus far, we have reviewed the ways groups of edges form constructs that can be used to probe the organization of a brain network. Groups of edges can capture patterns beyond the pairwise relationship reported by a single edge (Figure 3D). Another avenue for uncovering such patterns is to employ the tools of algebraic topology (Battiston et al., 2020), which provide a formal mathematical framework for analyzing the higher order relational content of a network by using concepts such as cliques and cavities (Giusti, Pastalkova, Curto, & Itskov, 2015; Sizemore, Phillips-Cremins, Ghrist, & Bassett, 2019). Applied to brain data, such tools show how all-to-all components of a network may serve to localize hublike roles that some brain areas might play (Sizemore et al., 2018) or help to elucidate spiking activity progression in large neuronal microcircuit simulations (Nolte, Gal, Markram, & Reimann, 2020). An advantage of these approaches is the ability to describe how components of the ordinary network of pairwise relationships take part in higher order mesoscale organization, observable by applying mathematical reformulations like filtrations. Applications have highlighted the increase in integrative organization after administration of psychoactive drugs like psilocybin by identifying edges that support topological cycles (Petri et al., 2014). Algebraic topology also offers new ways to draw relationships between nodes based on clustering in a low-dimensional embedding space (Patania et al., 2019).

Without edges, a network would merely be a set of nodes with no relational content. All network assessments, even the ones that produce node-wise measurements like clustering coefficient, need edge data. Evidently, edges are trivially important for network analysis. This section highlighted the further utility of edge groupings to understand levels of organization in brain networks. These approaches complement other methods like psychophysiological interaction analysis (O'Reilly, Woolrich, Behrens, Smith, & Johansen-Berg, 2012) or bundle analysis (Chandio et al., 2020), which provide ways to extract rich multivariate data about interareal relationships outside of a network context. Overall, the complex structural and functional organization of the brain can be explored through relational information. In particular, the features that form from groups of edges, from motifs to fingerprints to communities and cliques establish local relationships that enable specific functional capabilities or place nodes within a global connectivity context.

## EDGES IN COMMUNICATION AND BRAIN DYNAMICS

The history of neuroscience provides us with vast cumulative knowledge about the localization of structural and functional features across the cortex and subcortex, from the micro- to the macroscale, resulting in comprehensive maps of the brain (Amunts & Zilles, 2015; Poldrack & Yarkoni, 2016). Through extensive brain mapping studies, specific areas can be associated with specialized function, tuned to a behavior or cognitive processes. Such maps document the spatial layout of areas, but not necessarily how these areas interact. The addition of edges to a map provides information about how the elements of a map collectively form an integrative system, supportive of both local and distributed activity. Edges are also key for studying brain communication. They can represent the structural scaffold on which communication unfolds and channel the ongoing dynamic activity between neural elements (Avena-Koenigsberger et al., 2018). Here we examine the role of edges, and information at the edges, for understanding how the brain forms an integrative communicating system.

### Structure-Function Relationships

A profitable starting point for investigating brain communication is to assess the relationship between structural and functional network organization (Bansal, Nakuci, & Muldoon, 2018; Suarez, Markello, Betzel, & Misic, 2020), to observe the extent to which structural edge weights estimated in vivo possibly constrain the resultant functional topology. Focusing on edge weights, we can find a moderate positive association between structure and function at group and individual levels in humans (Honey et al., 2009; Zimmermann, Griffiths, Schirner, Ritter, & McIntosh, 2018), across node sets (Messe, 2020), and even in other species including invertebrates (Turner, Mann, & Clandinin, 2021). However, the structure-function relationship is more complex than implied by an edgewise comparison—for example, it can be confounded by overlap and transitivity (Zalesky et al., 2012) and biased by distance (Honey et al., 2009). Notably, the communication that takes place between network nodes is a complex mixture of effects due to numerous intersecting paths (Avena-Koenigsberger et al., 2017). The observed statistical dependence at any one edge is a result of communication through direct connections and a mix of local and global contexts. Thus, structure-function relationships may be better modeled by utilizing information beyond the pairwise connectivity. Take for example, the comparison of structural and functional connectivity fingerprint coupling at each node (Baum et al., 2020; Vázquez-Rodriguez et al., 2019), which follow smooth gradients of functional topography. Other sorts of higher order contexts, such as embedding vectors generated from biased random walks on the structural network (Levakov, Faskowitz, Avidan, & Sporns, 2021; Rosenthal et al., 2018), can predict the functional topology with greater accuracy.

**Figure 4.**
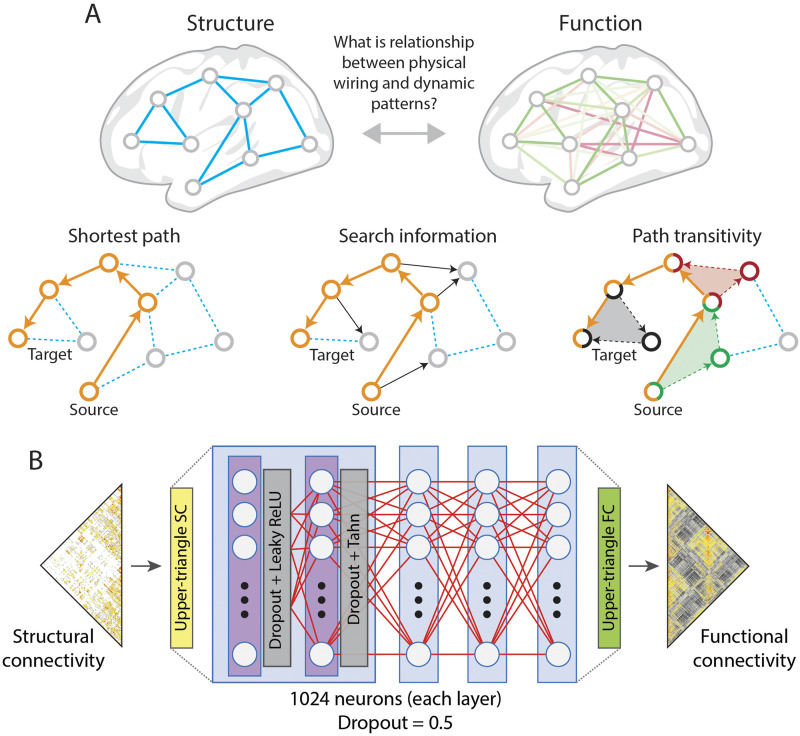
Edges can report both anatomical and functional relationships between regions. (A) How these two topologies relate to each other remains an important topic of investigation for network neuroscience; one way to approach this question is to model how communication processes, guided by certain algorithmic rules, might unfold over the structural edges. In shortest paths routing, communication between a source and target node unfolds along the shortest structural path. From the perspective of a diffusion process or knowledgeless random walker, accessing the shortest path may be difficult if there exist opportunities for the walker to “hop” off the path (we show these opportunities as black arrows in the middle panel). The total information (usually expressed in units of bits) required to navigate the shortest path successfully is referred to as “search information.” Even if a random walker diverges from the shortest path, there may be opportunities to return. This intuition is quantified by the measure “path transitivity,” which identifies cases where, following deviations from a network’s shortest path, a random walker can return. In the figure, we highlight three such cases, plotting the deviation and return as a filled triangle (red, black, and green). (B) The mapping between structure and function can also be estimated via deep learning, which can achieve high prediction accuracies at the group and individual levels (Sarwar, Tian, Yeo, Ramamohanarao, & Zalesky, 2021). Although such an approach cannot uncover putative neurophysiological mechanisms, the model performance can serve as a benchmark for other types of structure-function mapping models (figure adapted from Sarwar et al., 2021).

Since structural edges may provide a scaffold on which communication takes place (Figure 4A), it makes sense that network communication modeling has been taken up by neuroscientists to explain structure-function relationships. Many communication models are based on network paths over a topology that is assumed to be efficiently wired, based on metabolic and volumetric constraints. Communication models based on paths taken over the structural topology produce edgewise information about the ease of communication between nodes, for example, diffusion (Abdelnour, Voss, & Raj, 2014), search information (Goni et al., 2014), and navigability (Seguin, van den Heuvel, & Zalesky, 2018; Vázquez-Rodriguez, Liu, Hagmann, & Misic, 2020). These coefficients, or combinations thereof, can predict (or correlate with) the functional topology. The incorporation of higher order information, or polysynaptic signaling, not only improves alignment with the empirical functional topology, but also increases the predictive utility of structural connectivity, allowing for better prediction of broad behavioral dimensions (Seguin et al., 2020).

Understanding the mapping from structure to function has been scrutinized using frameworks ranging from communication modeling (Avena-Koenigsberger et al., 2018) to deep learning (Sarwar et al., 2021) to neural mass modeling (Sanz-Leon, Knock, Spiegler, & Jirsa, 2015) (Figure 4B). In this pursuit, the target goal is made more difficult by the fact that most pairwise estimates of dynamic interaction, communication, or functional connectivity are averaged over time. Time-averaged estimates of functional similarity could be insensitive to important dynamics at the edge level that reflect communication processes. Therein lies a motivation for observing edgewise and time-resolved functional connectivity.

### Time-Varying Functional Connectivity

We expect that communication between brain regions would ebb and flow over short timescales, reflected in a sequence of correlation or coupling values at each edge. These dynamics could be in response to varying cognitive demands and environmental cues or reflect a dynamic repertoire of intrinsic functionality. Recent emphasis has been placed on tracking and quantifying how functional coactivation changes moment by moment between nodes, termed dynamic or time-varying functional connectivity (Lurie et al., 2020). In practice, time-varying connectivity resolves the transient relationships between regions, which can signal different internal states that the brain is occupying or passing through (Fukushima et al., 2018). These dynamics are driven by external stimuli (Simony et al., 2016) and are associated with clinical grouping or outcome (Douw et al., 2019) or patterns of structural topology (Fukushima & Sporns, 2020; K. Shen, Hutchison, Bezgin, Everling, & McIntosh, 2015; Zamora-Lopez, Chen, Deco, Kringelbach, & Zhou, 2016).

There are two main approaches for studying time-varying connectivity, using either model-based dynamical systems that simulate the activity of neural populations, or data-driven statistical evaluations that operate on the observed time series (Lurie et al., 2020). A common data-driven method for rendering dynamic correlation values is by subdividing the empirical time series into many overlapping windows. For each window, a correlation matrix is calculated, generating a sequence of values at each edge representing changing coactivity from window to window. Such an approach is subject to key parameter choices, like window length and offset (Shakil, Lee, & Keilholz, 2016) that can affect the detection or potentially blur sharp or instantaneous periods of synchrony.

### Edge Time Series

Recently, a new approach has been proposed that obviates the need for sliding windows, while recovering a frame-by-frame account of an edge’s activity (Faskowitz, Esfahlani, Jo, Sporns, & Betzel, 2020; Zamani Esfahlani et al., 2020). An edge time series is constructed by multiplying the *z-*scored signals of two nodes, which also happens to be an intermediate step of calculating Pearson’s correlation (van Oort et al., 2018). These time series track each edge’s functional cofluctuations at the same temporal resolution as the original signal. Applying this construct to fMRI data, we observe intermittent high amplitude “events” of cofluctuation that account for a large portion of the classic time-averaged functional connectivity. This finding implies that the time-averaged functional connectivity estimate is driven by brief epochs of burst-like activity (X. Liu & Duyn, 2013; Tagliazucchi, Balenzuela, Fraiman, & Chialvo, 2012; Thompson & Fransson, 2016). Interestingly, high amplitude frames reflect a shared functional organization, and yet, also exhibit deviations to reliably distinguish subjects from each other (Betzel, Cutts, Greenwell, & Sporns, 2021). A further property of edge time series is that, at any given frame, the instantaneous cofluctuation pattern is partitioned into exactly two communities (Sporns, Faskowitz, Teixeira, Cutts, & Betzel, 2021). This feature implies that canonical functional systems are only transiently expressed, and that their familiar brain-wide architecture results from the superposition of many bipartitions over time. Future edge time series work should focus on disambiguating dynamic properties from time-invariant properties of the data, which can be explained with null models that incorporate the pairwise covariance structure of the data (Novelli & Razi, 2021).

By recovering temporally resolved time series for each edge, the communication dynamics can be studied with high precision. The simple Pearson correlation “unwrapping” procedure can readily be extended to domains beyond fMRI such as electrophysiological recordings. Such recordings afford much higher sampling rates and could be analyzed with a variant of the edge time series that adds lag terms and hence could possibly establish directionality of the edge dynamics. In a further extension, at the neuronal level, models of spike transmission at the edge (synapse) level can be built (McKenzie et al., 2021). Additionally, mutual information can be “unwrapped” into pointwise mutual information that can also record time-resolved edge fluctuations (Lizier, 2014). Findings based on edge time series complement previous map-based approaches (X. Liu & Duyn, 2013), which also focus on the cofluctuating activity at single frames. Additionally, edge time series likely relate to the dynamic information that can be computed at the edge level via the Multiplication of Temporal Derivatives method, which has demonstrated increased temporal sensitivity to simulated and task-evoked changes in connectivity (Shine et al., 2015). There remains much to be explored regarding the networked edge dynamics, including the ongoing topology these dynamics form (Betzel et al., 2021) and the cofluctuation patterns that might evolve intrinsically (Lindquist, Xu, Nebel, & Caffo, 2014) or evoked during experimental manipulations (Cooper, Kurkela, Davis, & Ritchey, 2021; Rosenthal, Sporns, & Avidan, 2017).

## FUTURE DIRECTIONS

### Relationships Between Edges

**Figure 5.**
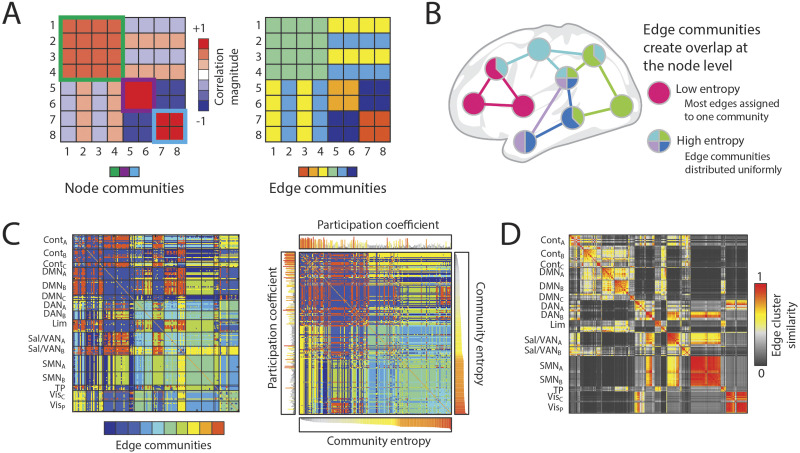
Edge-centric approaches allow for edges to be clustered directly, which can reveal mesoscale organization at the edge level. (A) Node-based clustering results in groupings of nodes that are commonly modular, and can be visualized as dense squares on the diagonal of an adjacency matrix; edge-based clustering results in groupings of edges with a common property, and can be visualized by coloring the adjacency matric with community affiliation. (B) Edge community overlap can be indexed by a node-level measurement of entropy, which characterizes the distribution of discrete communities connecting to each node. (C) A 10-community clustering of edge functional connectivity visualized as an adjacency matrix (left), and sorted by community entropy (right); the sorted matrix displays a “banding” pattern, which demonstrates a difference between high- and low-entropy nodes (figure adapted from Faskowitz et al., 2020). (D) The edge cluster similarity matrix indicates the similarity of edge community profiles, which are rows (or columns) of the edges community matrix (as in C); this matrix indicates the varying levels of edge community diversity contained within canonical functional systems (figure adapted from Faskowitz et al., 2020).

The common conceptualization of brain networks follows a familiar formula, which we have reviewed here, with *N* nodes describing the physical neural elements and the *E* edges describing the web of various types of interrelationships between these elements (Figure 5A). In this approach, we take the neural elements to be the fundamental units, to be compared in a pairwise manner (but see Box 3). An alternative approach would be to take the *edges* as the fundamental units (Ahn et al., 2010), to construct edge-edge matrices that index the similarity between edge information, particularly over time (Bassett, Wymbs, Porter, Mucha, & Grafton, 2014; Davison et al., 2015; Faskowitz et al., 2020; see also Iraji et al., 2016).

**Box 3.**  Parcellating nodes or edges

Even in a review of brain network edges, issues concerning the identification of nodes are worth noting. Edges are inexorably linked to nodes, documenting the relationship between the distinct elements of the neural system. The demarcation of neurons, neuronal populations, or cortical regions that constitute neural elements can be done using a range of methods (de Reus & Van den Heuvel, 2013). A change in the definition of nodes will likely necessitate that the edges be recomputed. Early studies dividing the cortex based on neuronal tissue properties continue to influence present-day cortical mapping (Amunts & Zilles, 2015). Other definitions of neural elements rely on the extraction of functionally coherent elements, such as the estimation of single units from an electrode array data (Dann, Michaels, Schaffelhofer, & Scherberger, 2016) or the grouping of spatially coherent and similarly active time series, ranging from the level of neurons to cortical vertices or voxels (Arslan et al., 2018; Genon et al., 2018). Altogether, these methods describe how neural data can be parcellated, resulting in a set of nodes.

While the history of neuroscience is riddled with attempts to create nodal parcellations or maps of cortex (Finger, 2001), considerably less attention has been devoted to defining or delineating distinct edges, for example, tracts of the white matter. Commonly, features mapped in (cytoarchitectonics) or onto (connectivity) the cortex and subcortex are used as inputs for parcellation methods, which are essentially applications of node-based clustering and segmentation. However, it is also possible to cluster and segment data that relates directly to edges, specifically signals from the brain’s white matter. For example, the streamline paths that result from tractography can be submitted to a hierarchical clustering routine, to create larger streamline groupings called bundles (Chandio et al., 2020; Garyfallidis, Brett, Correia, Williams, & Nimmo-Smith, 2012). Segmented tracts, when taken as fundamental building blocks of a network model, can be assembled into a matrix that records their intersections on cortical gray matter nodes. In such a model, tracts may be interpreted as conduits of specialized information or communication patterns that form elements of information processing (Pestilli, 2018). In another example, bold oxygen level–dependent signal in the white matter can be clustered, forming parcels that relate to canonical systems found in the gray matter (Peer, Nitzan, Bick, Levin, & Arzy, 2017). These examples demonstrate alternative ways in which “edge” information could be conceptualized as neural elements. While little has been done so far, such an approach seems promising as it leads us to reconsider the primary importance of cortical nodes and may stimulate further modeling of organization found within the white matter.

Comparing the pairwise temporal cofluctuation profiles of edges enables the creation of hyperedges, to reveal temporally similar edge bundles that evolved in a task-specific manner (Davison et al., 2015). These profiles can also serve as the basis of intersubject dynamic similarity evaluated during a movie watching task, which can flow between integrated and segregated topologies related to stimulus properties (Betzel, Byrge, Esfahlani, & Kennedy, 2020) or serve as the basis to investigate higher order correlations related to narrative content (Owen, Chang, & Manning, 2019). Comparing edge time series in a pairwise fashion results in an edge functional connectivity (eFC) matrix (Faskowitz et al., 2020). Clustering this matrix exposes a pervasively overlapping community structure (Figure 5B–5D) at the node level that not only bridges canonical systems, but also reveals nested edge-level structure for diverse canonical systems like the control and default mode network (Jo et al., 2020). Edge functional connectivity may also contain new sources of individual variation (Jo, Faskowitz, Esfahlani, Sporns, & Betzel, 2021). Taken together, these approaches suggest that taking the edges as fundamental network components provides a new perspective through which to interrogate brain organization.

### White and Gray Matters

The white matter is the anatomical tissue that, by volume, comprises over half of the human brain. In terms of interareal connectivity, the *white matter matters* (Fields, 2008). The dogma that the white matter is “passive wiring” is being challenged by evidence that the myelin plays a role in how action potentials are propagated through the brain, which in turn could affect oscillatory activity in the cortex (Fields, Woo, & Basser, 2015). At a macroscopic level, lesions in the white matter have been linked to specific object-naming deficits, suggesting a role for white-matter tracts in semantic knowledge (Fang et al., 2018; Pestilli, 2018). New methods are emerging that link cortical functional activity with white matter tracts (O'Muircheartaigh & Jbabdi, 2018; Tarun, Behjat, Bolton, Abramian, & Van De Ville, 2020), shedding light on how structural architecture might mediate macroscale dynamics or influence information flow. Furthermore, indices of white-matter integrity have long been linked with clinical deficits, suggesting a possible role for white matter in disease models (Hanekamp et al., 2021; Karlsgodt, 2020). These studies suggest that the white matter has the potential to shape dynamics and impact cognitive processing.

The brain network model is in part useful because it abstracts the complex geometry and biology of the brain into a simple mathematical representation. When visualizing networks, often edges are represented as straight lines through space, with thicknesses or transparency that denotes edges strength. However, we should not lose sight that this representation is divergent from the anatomical reality of the brain, which is embedded in space and contains topographically organized white-matter connections (Jbabdi et al., 2015; Kurzawski, Mikellidou, Morrone, & Pestilli, 2020). Structural edges travel along physical paths through the white matter that have shape, curvature, and volume, and that compete for physical space and limited metabolic resources. Similarity of functional activity could be influenced by activity-dependent myelination (Fields et al., 2015), or possible ephaptic coupling of sheets of axons within white-matter tracts (Sheheitli & Jirsa, 2020). Thus, future work along these lines should focus on better understanding how the white matter plays a role in differentially shaping the relational content of brain networks.

Although the edges we model often represent the macroscopic interareal pathways that pass through white matter, at the microscale neural signals also propagate locally within the gray matter (Voges, Schüz, Aertsen, & Rotter, 2010). Local and recurrent connectivity is commonly a parameter in neural mass or field models, serving to enrich the repertoire of spatiotemporal dynamics (Proix et al., 2016) and used to situate cortical regions along a functional hierarchy (Wang et al., 2019). Although common imaging acquisitions might not resolve this microscale architecture directly, such relationships can be added to brain network models. Edges representing spatial adjacency can be added to a brain network to account for presumed local influences. Doing so can enhance the modeling of functionally meaningful connectome harmonics patterns on cortical surface (Naze, Proix, Atasoy, & Kozloski, 2021). The intrinsic curvature of the cortical surface mesh, which is influenced by differential growth patterns, has been proposed as a marker of local connectivity that could serve as an edge weight (Ecker et al., 2013; Ronan et al., 2012). Furthermore, with advances in imaging techniques and acquisitions, high-resolution data will be better suited to render the fine-grained architecture of the gray matter, enabling the observation of tangential fibers (Leuze et al., 2014) and the estimation of microstructural indices (Fukutomi et al., 2018). Finally, future investigations could benefit from bridging scales of observation, by incorporating cytoarchitectural indices or transcriptomic information (Paquola et al., 2020) to inform macroscale edge weights. Going forward, modeling these local relationships that are commonly unaccounted for could enhance brain network model fidelity and, in turn, aid in our understanding of structure-function relationships.

### Subject-Specific Edge Information

Recent emphasis has been placed on extracting information from fMRI functional connectivity data, to characterize organizational features that robustly associate with a specific trait, like intelligence or attention (Finn et al., 2015; Rosenberg et al., 2016; X. Shen et al., 2017). This *connectome predictive modeling* approach involves filtering edges based on statistical criteria (such as correlation with a phenotype) and summing the edge weights for each subject. These sums are then used to create a statistical prediction model, in left-out subject data. The resultant cross-validated model outlines a set of edges important for predicting a desired phenotype. Notably, the networked characteristics of these edges can be analyzed to reveal system-level organization, such as the number of between system edges that participate in a high-attention predictive model (Rosenberg et al., 2016). This approach demonstrates the potential for mapping brain-behavior correlations at the level of brain edges. It remains to be seen how these predictive models could be extended to utilize edge constructs that capture higher order relationships, which could be a productive future direction in tandem with the growing interest in applications of algebraic topology to brain network data.

## CONCLUSION

In contrast to brain network nodes, whose definition and differentiation have been the focus of brain mapping studies for years, issues and concepts relating to brain network edges have been less central to date. Here we have reviewed ways in which the edges matter, in terms of construction approaches that influence network analysis or in settings where groups of edges form higher order relational information available for analysis. Furthermore, edges are a prime candidate through which to explore how communication processes unfold within the brain. Regardless of data modality, across neural data that spans spatial and temporal scales, we advocate for careful consideration of the information at the edge level. A greater focus on the information contained at the edges, otherwise known as an edge-centric perspective (de Reus et al., 2014; Faskowitz et al., 2020), can potentially stimulate novel exploration of brain organization. Both nodes and edges are fundamentally intertwined as the basic ingredients of a network model. Network neuroscience explorations can evidently benefit from both edge-centric and node-centric perspectives.

## ACKNOWLEDGMENTS

We acknowledge the following manuscripts from which figures were adapted: Figure 2, de Reus et al. (2014) (with permission from The Royal Society, UK), Faskowitz et al. (2021); Figure 3, Z.-Q. Liu et al. (2020) (CC BY 4.0), Balsters et al. (2020) (CC BY 4.0), Betzel et al. (2018) (CC BY 4.0), Sizemore et al. (2018) (CC BY 4.0); Figure 4, Sarwar et al. (2021) (CC BY 4.0); Figure 5, Faskowitz et al. (2020).

## ROLE INFORMATION

Joshua Faskowitz: Writing – original draft; Writing – review & editing. Richard F. Betzel: Writing – original draft; Writing – review & editing. Olaf Sporns: Writing – original draft; Writing – review & editing.

## FUNDING INFORMATION

Joshua Faskowitz, National Science Foundation (https://dx.doi.org/10.13039/100000001), Award ID: 1342962. Richard F. Betzel, National Science Foundation (https://dx.doi.org/10.13039/100000001), Award ID: 2023985. Olaf Sporns, National Science Foundation (https://dx.doi.org/10.13039/100000001), Award ID: 2023985. Olaf Sporns, National Institute of Mental Health (https://dx.doi.org/10.13039/100000025), Award ID: 5R01MH122957. Joshua Faskowitz, Indiana University Bloomington (https://dx.doi.org/10.13039/100010178), Award ID: Dissertation Research Fellowship. Richard F. Betzel, Indiana University Bloomington (https://dx.doi.org/10.13039/100010178), Award ID: Emerging Area of Research Initiative.

## TECHNICAL TERMS

Parcellation: The demarcation of distinct neural elements based on qualitative or quantitative criteria collected from structural and functional brain data.
Topographically organized: A description of the ordered spatial arrangement of neuroanatomical tissue.
Cytoarchitectonic: Describing the composition of neural tissue at the microscale, relating to the distribution, density, size, and shape of neural cell bodies.
Correlated gene expression: The similarity of gene transcription and/or translation levels between distinct samples of neuroanatomical tissue.
Structural covariance: A measure of the tendency for neuroanatomical indices to covary, commonly applied to brain measurements across individuals of a group.
Informatic collation: The synthesis of experimental evidence gathered from a database of the relevant scientific literature.
Self-loops: An edge that connects a node to itself, i.e., the diagonal of an adjacency matrix.
Hyperedge: An edge that can link an arbitrary number of nodes, thereby describing relationships that are not necessarily pairwise.
Minimum spanning tree: The set of edges with minimal weight/cost that connects all nodes; concept can be analogously applied to find the maximum spanning tree.
Overlapping community structure: A clustering of elements in which each element can be affiliated with one or more clusters or communities.
